# Letter to the Editor

**DOI:** 10.1179/107902611X13046044199129

**Published:** 2011-05

**Authors:** James H. Frisbie

**Affiliations:** St. Paul, Minnesota

**To the editor:**

**Re: Weaver FM, *et al*. J Spinal Cord Med. 2011;34(1):35–45. Smoking behavior and delivery of evidence based care for veterans with spinal cord injuries and disorders.**

Weaver *et al.*^1^ have described the high prevalence, 73%, of smoking among veterans at the time of spinal cord injury. Noting that less than half who abstained in a 20 year follow-up did so during their initial hospitalization, they suggest that providers counsel their patients about smoking during the rehabilitation program. Some data that can reinforce this suggestion can be cited.^2^ In an average follow-up of 18 years by interview we also found that fewer than half (23 of the 55 veterans) who quit smoking, did so within 1 year of injury. These early abstainers cited health concerns raised by themselves or family or a loss of taste for cigarettes during the rehabilitation period. It appears, as Weaver *et al.* have suggested, that following a life changing event such as spinal cord injury or dysfunction, there exists a particular opportunity for the rehabilitation provider to help in the abstinence from smoking.
